# Crystal structure and Hirshfeld surface analysis of the hydrated 2:1 adduct of piperazine-1,4-diium 3,5-di­nitro-2-oxidobenzoate and piperazine

**DOI:** 10.1107/S2056989022000226

**Published:** 2022-01-14

**Authors:** Veerappan Subha, Thangaraj Seethalakshmi, Thangavelu Balakrishnan, M Judith Percino, Perumal Venkatesan

**Affiliations:** a PG and Research Department of Physics, Government Arts College (Autonomous and affiliated to Bharathidasan University, Tiruchirappalli), Thanthonimalai, Karur-639 005, Tamil Nadu, India; b Crystal Growth Laboratory, PG and Research department of Physics, Periyar EVR Government College (Autonomous and affiliated to Bharathidasan University, Tiruchirappalli), Tiruchirappalli-620 023, Tamil Nadu, India; c Unidad de Polímeros y Electrónica Orgánica, Instituto de Ciencias, Benemérita Universidad Autónoma de Puebla, Val3-Ecocampus Valsequillo, Independencia O2 Sur 50, San Pedro Zacachimalpa, 72960, Puebla, Mexico; d Department of Chemistry, Srimad Andavan Arts and Science College (Autonomous), Tiruchirappalli-620 005, Tamil Nadu, India

**Keywords:** crystal structure, 3,5-di­nitro­salicylate dianion, piperazine-1,4-diium cation, organic proton-transfer salt, Hirshfeld surface analysis

## Abstract

The crystal structure of the adduct piperazine-1,4-diium 3,5-di­nitro-2-oxidobenzoate–piperazine–water (2/1/2) shows the existence of a 3,5-di­nitro­salicylate dianion (DNSA^2−^) and a protonated piperazine-1,4-diium cation (PIP^2+^) along with a piperazine mol­ecule. The formula of the title adduct in the asymmetric unit is 2C_4_H_12_N_2_^2+^·2C_7_H_2_N_2_O_7_^2−^·C_4_H_10_N_2_·2H_2_O with *Z* = 1. The piperazine ring in the piperazine-1,4-diium cation and in the neutral piperazine mol­ecule adopt chair conformations. All O atoms in the DNSA^2−^ moiety and the water mol­ecule act as hydrogen-bonding acceptors for various inter­molecular O—H⋯O, N—H⋯O and C—H⋯O inter­actions, which stabilize the crystal structure. Various supra­molecular architectures formed by the different inter­molecular inter­actions are discussed. The relative contribution of various inter­molecular contacts is analysed with the aid of two-dimensional (full and decomposed) fingerprint plots, indicating that H⋯O/O⋯H (50.2%) and H⋯H (36.2%) contacts are the major contributors to the stabilization of the crystal structure.

## Abstract 2

In the title adduct, two 3,5-di­nitro­salicylic acid mol­ecules in the dianionic (DNSA^2−^) form, two protonated piperazine-1,4-diium cations (PIP^2+^) and a neutral piperazine mol­ecule (PIP) along with two water mol­ecules are found in the asymmetric unit. The crystal structure of the title adduct is reported, and hydrogen-bonding inter­actions are discussed.

## Chemical context

3,5-Di­nitro­salicylic acid (DNSA) is one of the most prevalent proton-donor mol­ecules for forming organic salts with different Lewis bases. There are more than 150 examples found in the Cambridge Structural Database (CSD, Version 5.41, update of August 2020; Groom *et al.*, 2016 ▸) containing the DNSA moiety. Among them, there are 20 structures containing the neutral DNSA mol­ecule with the rest being proton-transfer salts of the monoanion DNSA^−^ and the dianion DNSA^2−^. The loss of both acidic protons from the carb­oxy­lic acid (–COOH) and phenolic (–OH) groups in the DNSA mol­ecule forms the DNSA^2−^ dianion. In general, the removal of the acidic proton from the –COOH group in DNSA [p*K*_a_(COOH) = 2.2] would be expected to occur more readily than the removal of the proton from the phenolic –OH group [p*K*_a_(OH) = 6.8]. Consequently, the DNSA mol­ecule easily forms 1:1 proton-transfer salts with aliphatic amines (Smith *et al.*, 2002 ▸), monocyclic, polycyclic aromatic and heteroaromatic amines (Smith *et al.*, 2003 ▸, 2007 ▸), substituted primary and secondary anilines and phenyl­enedi­amines (Issa *et al.*, 1980 ▸, 1981 ▸; Hindawey *et al.*, 1980 ▸). However, 1:2 proton-transfer salts of DNSA containing the DNSA^2−^ dianion are much fewer in number. Briefly, the structurally characterized 1:2 proton-transfer salts were formed with ethyl­enedi­amine (EGUVAD; Smith *et al.*, 2002 ▸), cyclo­hexyl­amine (ROFLIJ; Gao *et al.*, 2014 ▸), piperidine (XEBFAM; Smith *et al.*, 2006 ▸) and di­ethyl­enetri­amine (ZONBIP; Chen *et al.*, 2014 ▸). Among the four DNSA^2−^ salts, the asymmetric unit of ROFLIJ consists of two cyclo­hexyl­aminium cations and a DNSA^2−^ moiety while one dication (di­ethyl­enetriaminium dication in ZONBIP or ethyl­enediaminium dication in EGUVAD), DNSA^2−^ and one water mol­ecule are found in the asymmetric unit of ZONBIP or EGUVAD. The dianions (DNSA^2−^), mono anions (DNSA^1−^), and partially substituted mono picrate anion along with three piperidinium cations and a water mol­ecule are found in the asymmetric unit of XEBFAM. The crystal structures (EGUVAD, ROFLIJ, ZONBIP, and XEBFAM) of these DNSA^2−^ salts are mainly stabilized by N—H⋯O, C—H⋯π and π–π inter­actions. On the other hand, co-crystals of DNSA were reported with phenazine (Senthil Kumar *et al.*, 2002 ▸), urea or substituted ureas (Smith *et al.*, 1997 ▸, 2000 ▸; Bott *et al.*, 2000 ▸) and *trans*-1,4-di­thiane-1,4-dioxide (Senthil Kumar *et al.*, 2002*b* ▸). In this study, the crystal structure, Hirshfeld surface (HS) analysis, structural features, and various inter­molecular inter­actions that exist in the monohydrated 1:1 adduct of bis­(piperazine-1,4-diium) 3,5-di­nitro-2-oxidobenzoate and piperazine (I) are reported. The various inter­molecular inter­actions and the relative contribution of various inter­molecular contacts are compared with a similar structure (XEBFAM). 
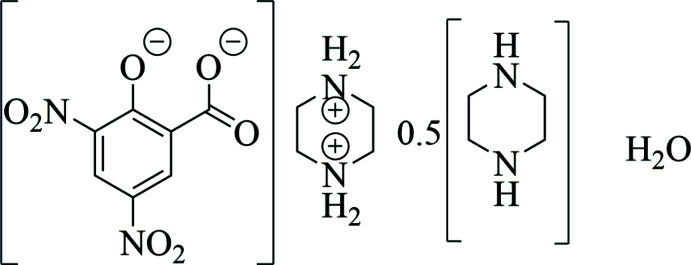


## Structural commentary

**Figure 1:**
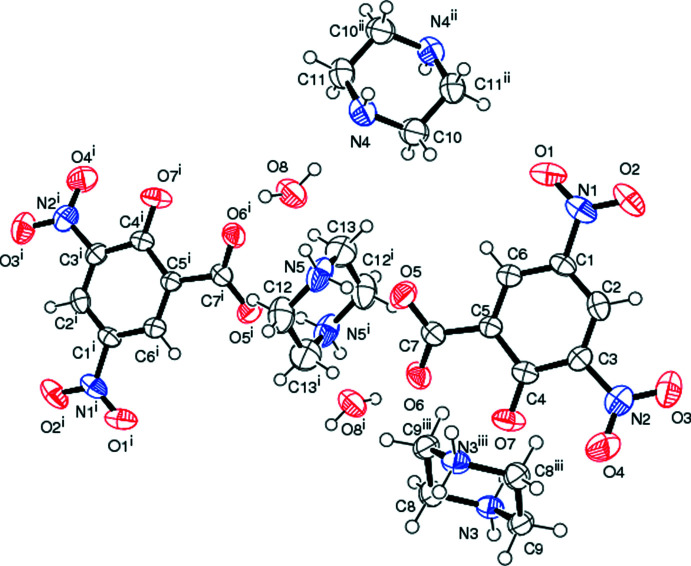
The mol­ecular structure of the title adduct, (I), showing the atom-labelling scheme [symmetry codes: (i) −*x* + 2, −*y* + 2, −*z* + 2; (ii) −*x* + 1, −*y* + 1, −*z* + 2; (iii) −*x*, −*y* + 2, −*z* + 1]. Displacement ellipsoids are drawn at the 50% probability level.

The title compound crystallizes in the triclinic space group *P* 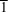 with *Z* = 1 with the asymmetric unit comprising two DNSA^2−^ ions, two protonated piperazine-1,4-diium cations, and a neutral piperazine mol­ecule along with two water mol­ecules and having the formula 2C_4_H_12_N_2_^2+^·2C_7_H_2_N_2_O_7_^2−^·C_4_H_10_N_2_·2H_2_O. The atom-numbering scheme and mol­ecular structure of (I) are shown in Fig. 1 ▸. The distance between the phenolate oxygen atom, O7, and the carboxyl­ate oxygen atom, O6, in the anion is 2.770 (2) Å and is comparable to that found earlier reported dianionic salts (2.735–2.912 Å). This, together with the absence of a locateable H atom between these oxygen atoms (O6 and O7) is good evidence for the existence of the dianion in this adduct. One of the nitro groups (N1, O1, and O2) and the phenolate oxygen atom, O7 are coplanar with the mean plane of the phenyl ring, while the second nitro group (N2, O3, and O4) and the carboxyl­ate group (C7, O5, and O6) are slightly twisted from the above plane. These twists are measured by the dihedral angles between the mean plane of the phenyl ring and those of the second nitro and carboxyl­ate groups of 19.4 (3) and 24.4 (3)°, respectively, and by the C2—C3—N2—O4 and C6—C5—C7—O6 torsion angles of 161.1 (2)° and 156.6 (2)°, respectively. These slight twists of the nitro and carboxyl­ate groups are due to the differences in the inter­molecular hydrogen-bonding patterns in which the N2/O3/O4 and C7/O6/O7 groups participate as compared to the N1/O1/O2 group.

The piperazine rings in the piperazine-1,4-diium cations and the neutral piperazine mol­ecule in (I) adopt chair conformations with puckering parameters (Cremer & Pople, 1975 ▸), *Q* = 0.563 (3) Å, θ = 180.0 (3)°,φ = 0° for the the N3 ring, *Q* = 0.571 (3) Å, θ = 1.87 (1)°, φ = 0° for the N5 ring and *Q* = 0.517 (3) Å, θ = 180.0 (3)°, φ = 0° for the N4 ring in the neutral PIP mol­ecule.

**Figure 2:**
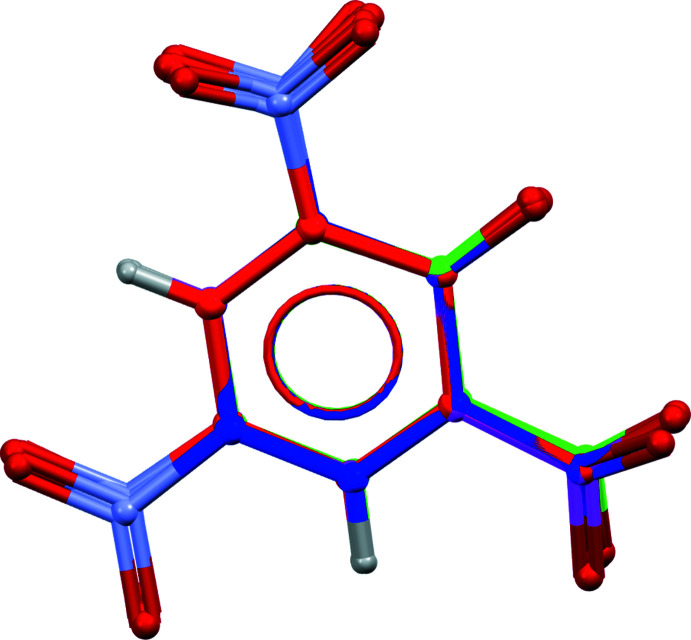
Superimposition of DNSA^2−^ units in (I) and its analogs [colour codes: EGUVAD (green), ROFLIJ (blue), ZONBIP (red) and XEBFAM (magenta)].

Additionally, we carried out a structural overlay study of the DNSA^2−^ units in the di-anionic salts found in (I), EGUVAD (Smith *et al.*, 2002 ▸), ROFLIJ (Gao *et al.*, 2014 ▸), ZONBIP (Chen *et al.*, 2014 ▸) and XEBFAM (Smith *et al.*, 2006 ▸) using the six carbon atoms in the phenyl ring in DNSA^2−^ as the basis. The DNSA^2−^ units in all five structures overlay quite well with one another. The maximum r.m.s.d. observed between any mol­ecular pair is 0.0095 Å (for ROFLIJ and ZONBIP). However, the slight rotation of the nitro and carboxyl­ate groups in the DNSA^2−^ unit (Fig. 2 ▸) may well be due to the oxygen atoms in these functional groups participating in different inter­molecular inter­actions in their crystal structures as noted above.

In the unit cell, two piperazine-1,4-diium cations and one DNSA^2−^ anion are linked *via* N3—H3*B*⋯O7, N3—H3*B*⋯O4 and N5—H5*B*⋯O5 hydrogen bonds (Table 1 ▸). Furthermore, the second piperazine-1,4-diium cation is linked to a piperazine mol­ecule through a water mol­ecule *via* N5—H5*A*⋯O8 and O8—H8*C*⋯N4 hydrogen bonds.

## Supra­molecular features

**Figure 3:**
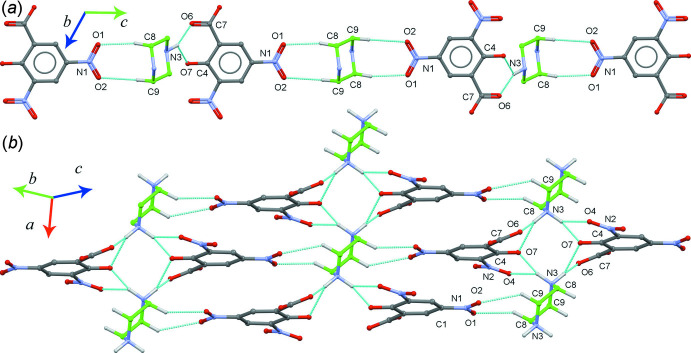
(*a*) Part of the crystal structure of (I) showing the 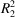 (8) and 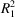 (6) motifs formed by inter­molecular N—H⋯O and C—H⋯O hydrogen bonds (see Table 1 ▸), which link the neighbouring anionic unit (DNSA^2−^) and cationic moiety (PIP^2+^) into a mol­ecular chain, which propagates parallel to the *c* axis. (*b*) Part of the crystal structure of (I) showing the sheet-like architecture.

**Figure 4:**
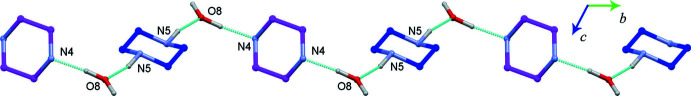
Part of the crystal structure of (I) showing the mol­ecular chain, formed by O8–H8*C*⋯N4 and N5—H5*A*⋯O8 hydrogen bonds, which propagates parallel to the *b* axis.

**Figure 5:**
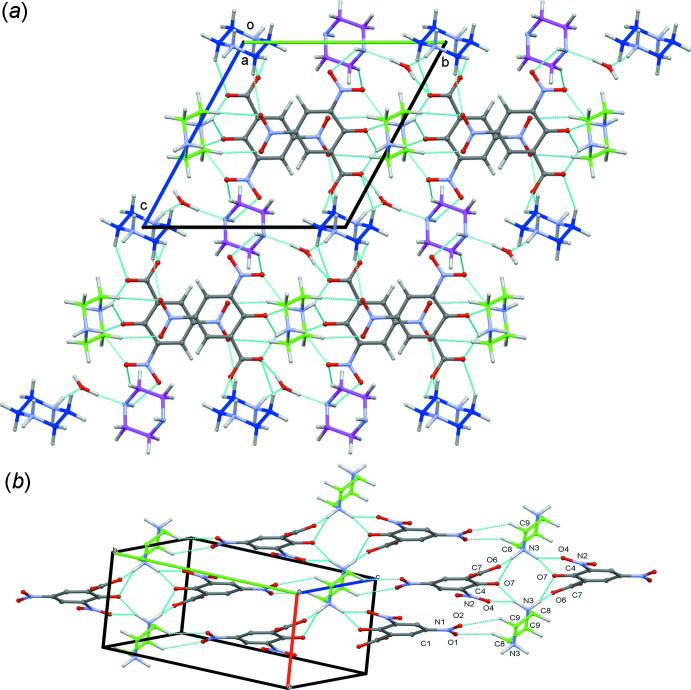
Crystal packing of (I), (*a*) viewed along the *a* axis and (*b*) viewed along the *c* axis.

The O1–O4 oxygen atoms in both nitro groups and the oxygen atoms in the carboxyl­ate (O6 and O7) and the phenolate groups (O8) in the DNSA^2−^ ion act as acceptors for various inter­molecular N—H⋯O and C—H⋯O inter­actions (Table 1 ▸). The atoms O1 and O2 in one nitro group form C8—H8*A*⋯O1 and C9—H9*B*⋯O2 hydrogen bonds, which link neighbouring DNSA^2−^ and PIP^2+^ ions with 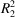 (8) motifs. The O6 and O7 atoms (from phenolate and carboxyl­ate groups) form a cyclic bidentate hydrogen bond with the H3*A*—N3 unit N3—H3*A*⋯O6 and N3—H3*A*⋯O7) with an 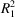 (6) motif. This 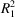 (6) motif is a common one in proton-transfer compounds of DNSA and it helps to extend their secondary structures (Smith *et al.*, 2007 ▸). These two ring motifs [ 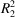 (8) and 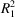 (6)] link a DNSA^2−^ anion and one of the PIP^2+^ cations into a mol­ecular chain, which propagates parallel to the *c* axis (Fig. 3 ▸ *a*). Furthermore, N3—H3*A*⋯O6, N3—H3*A*⋯O7, N3—H3*B*⋯O4 and N3—H3*B*⋯O7 inter­actions link two neighbouring mol­ecular chains through the PIP^2+^ cations into a sheet-like architecture containing two 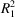 (6) motifs. Also involved is a weak C9—H9*B*⋯O2 inter­action (Fig. 3 ▸ *b*) which, although quite long, has precedent in recent work (Sosa-Rivadeneyra *et al.*, 2020 ▸). The water mol­ecule links the second piperazine-1,4-diium cation and a piperazine mol­ecule, PIP through O8–H8*C*⋯N4 and N5—H5*A*⋯O8 hydrogen bonds to form a mol­ecular chain (Fig. 4 ▸). Additional O—H⋯O, N—H⋯O and C—H⋯O hydrogen bonds produce a three-dimensional framework (Fig. 5 ▸).

## Hirshfeld surface analysis

**Figure 6:**
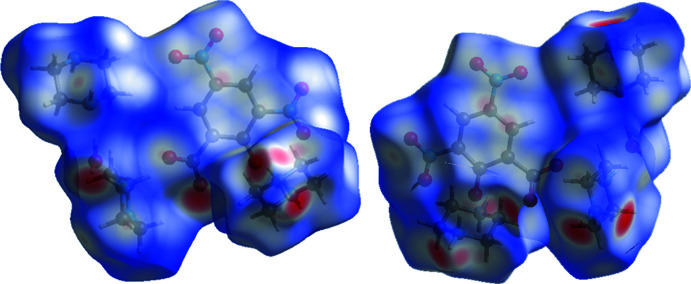
Two different views of the Hirshfeld surface of (I).

**Figure 7:**
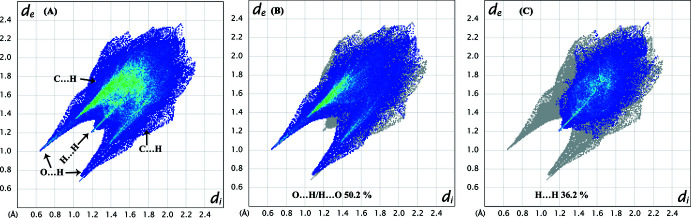
The two-dimensional fingerprint plots for (I): (*A*) a complete unit (various types of contacts are indicated); (*B*) H⋯H contacts and (*C*) O⋯H/H⋯O contacts.

*Crystal Explorer 17.5* (Turner *et al.*, 2017 ▸) was used to calculate the Hirshfeld surfaces (HS; McKinnon *et al.*, 1998 ▸, 2004 ▸) of the title adduct and generate two-dimensional fingerprint plots (full and decomposed, 2D-FP; Spackman & McKinnon, 2002 ▸; Spackman & Jayatilaka, 2009 ▸). The HS and 2D-FP were used to provide additional information and to qu­antify the inter­molecular inter­actions using distinct colours and intensities to indicate short and long contacts, as well as the relative contribution of the different inter­actions in the solid-state (Venkatesan *et al.*, 2015 ▸, 2016 ▸). The HS is plotted over *d*_norm_ in the range −0.7438 to 1.3459 a.u. and two views (front and back) of the HS are shown in Fig. 6 ▸. Bright-red spots on the HS confirm the existence of hydrogen-bonding contacts in the crystal structure. The 2D FP plots show that the relative contributions of the various non-covalent contacts (Fig. 7 ▸). O⋯H contacts contribute most (50.2%) to the crystal packing while the second significant contact is H⋯H, which contributes 36.2%. The relative contributions of C⋯O, C⋯H, N⋯H, C⋯N and C⋯C contacts are 4.6%, 2.9%, 2.7%, 1.7% and 1.0%, respectively. In the XEBFAM structure, the relative contributions of O⋯H, H⋯H, C⋯O, C⋯H, N⋯H, C⋯N and C⋯C contacts are 49.7%, 37.6%, 3.6%, 2.7%, 1.3%, 0.7%, and 2.1%, respectively. The relative contribution of various inter­atomic contacts in XEBFAM and the title adduct (I) are similar, even though the compounds have different compositions as discussed earlier.

## Database survey

A search of the Cambridge Structural Database (CSD, Version 5.41, update of August 2020; Groom *et al.*, 2016 ▸) using *Conquest* (Bruno *et al.*, 2002 ▸) for the neutral DNSA mol­ecule found 20 structures of co-crystals, including those with urea (NUHYAQ; Smith *et al.*, 1997 ▸), *trans*-1,4-di­thiane-1,4-dioxide (OGAHEJ; Senthil Kumar *et al.*, 2002*a* ▸), 4-(di­methyl­amino)­benzaldehyde (SUYYIW; Jin *et al.*, 2016 ▸) and dioxane (GORXAM, GORXAM01, GORXEQ, GORXEQ01; Senthil Kumar *et al.*, 1999 ▸). For monoanions of DNSA, a total of 62 structures containing the carboxyl­ate (COO^−^) moiety and 70 containing the phenolate anion (O^−^) were found. As mentioned earlier, the removal of the carb­oxy­lic acid proton is expected to be easier than the removal of the proton from the phenolic –OH group in DNSA so it is somewhat surprising that the number of crystal structures containing phenolate ions is larger than those containing carboxyl­ate ions. These seemingly conflicting results may suggest that the formation and stability of the salts with phenolate ions of the DNSA moiety is governed by inter­molecular inter­actions in the crystal. However, it has been pointed out (Fábry, 2018 ▸), that since the monoanions generally contain a hydrogen atom bridging between the the carboxyl­ate and phenolate oxygen atoms, how one formulates the anion (carboxyl­ate or phenolate) depends critically on how this hydrogen atom is treated in the refinement so that some of the reported phenolate structures may actually be carboxyl­ates.

As mentioned earlier, there are four structures of the dianionic salt of DNSA, which are formed with ethyl­enedi­amine (EGUVAD; Smith *et al.*, 2002 ▸), cyclo­hexyl­amine (ROFLIJ; Gao *et al.*, 2014 ▸), piperidine (XEBFAM; Smith *et al.*, 2006 ▸) and di­ethyl­enetri­amine (ZONBIP; Chen *et al.*, 2014 ▸). The cation (ethyl­enedi­amino­nium or 2,2′-imino­diethanaminium) and dianion (DNSA^2−^) along with a water mol­ecule are connected *via* inter­molecular O—H⋯O, N—H⋯O and C—H⋯O inter­actions in the asymmetric unit of EGUVAD and ZONBIP. An N—H⋯O hydrogen bond connects the cyclo­hexyl­amino­nium moiety and the dianion in ROFLIJ, while the piperazine-1,4-diium cations form a mixed salt with the dianion and monoanion of 3,5-di­nitro­salicylate along with a picrate anion in XEBFAM. The cation and anions are linked *via* O—H⋯O, N—H⋯O and C—H⋯O inter­actions in XEBFAM.

## Synthesis and crystallization

The title adduct was synthesized from 3,5-di­nitro­salicylic acid (1 mmol, 228 mg) and piperazine (5 mmol, 426 mg, 0.5 mL) dissolved in 50 mL of methanol and stirred well for 6 h. The homogeneous solution was filtered and the solution was allowed to evaporate slowly at room temperature. Red block-like crystals suitable for single X-ray diffraction were harvested after a growth period of 10 days.

## Refinement

Crystal data, data collection and structure refinement details are summarized in Table 2 ▸. The amino H atoms and O-bound H atoms were refined with DFIX instructions. The C-bound H atoms were included in calculated positions and treated as riding atoms: C—H = 0.93–0.98 Å, O—H = 0.82 Å with *U*_iso_(H) = 1.2*U*_eq_(C) and *U*_iso_(H) = 1.5*U*_eq_(O).

## Supplementary Material

Crystal structure: contains datablock(s) I, publication_text. DOI: 10.1107/S2056989022000226/mw2181sup1.cif

Structure factors: contains datablock(s) I. DOI: 10.1107/S2056989022000226/mw2181Isup3.hkl

CCDC reference: 2132861

Additional supporting information:  crystallographic information; 3D view; checkCIF report

## Figures and Tables

**Table 1:** Hydrogen-bond geometry (Å, °)

*D*—H⋯*A*	*D*—H	H⋯*A*	*D*⋯*A*	*D*—H⋯*A*
O8—H8*D*⋯O6^i^	0.84 (4)	1.98 (4)	2.807 (3)	173 (4)
N3—H3*A*⋯O6^ii^	0.90 (2)	1.92 (2)	2.752 (3)	153 (3)
N3—H3*A*⋯O7^ii^	0.90 (2)	2.33 (3)	2.940 (3)	125 (2)
N3—H3*B*⋯O4^iii^	0.90 (2)	2.50 (2)	3.142 (3)	129 (2)
N3—H3*B*⋯O7^iii^	0.90 (2)	1.84 (2)	2.696 (3)	157 (3)
N4—H4⋯O3	0.860 (17)	2.59 (2)	3.304 (3)	141 (2)
N5—H5*A*⋯O8	0.900 (17)	1.83 (2)	2.712 (3)	166 (3)
N5—H5*B*⋯O5^iv^	0.918 (17)	1.74 (2)	2.637 (3)	165 (3)
O8—H8*C*⋯N4	0.84 (4)	1.88 (4)	2.717 (3)	169 (3)
C8—H8*A*⋯O1	0.97	2.49	3.369 (3)	151
C9—H9*B*⋯O2	0.97	2.67	3.482 (3)	141
C12—H12*A*⋯O2^v^	0.97	2.58	3.455 (4)	151
C12—H12*B*⋯O5^i^	0.97	2.59	3.353 (4)	136

Symmetry codes: (i) -x+1, -y+2, -z+1; (ii) -x+1, -y+1, -z+1; (iii) x, y-1, z; (iv) x, y, z-1; (v) x+1, y+1, z.

**Table 2:** Experimental details

Crystal data	
Chemical formula	C_4_H_12_N_2_^2+^·C_7_H_2_N_2_O_7_^2−^·0.5C_4_H_10_N_2_·H_2_O
*M*_r_	375.35
Crystal system, space group	Triclinic, *P*\overline{1}
Temperature (K)	296
*a*, *b*, *c* (Å)	6.6211 (16), 11.891 (3), 12.389 (3)
α, β, γ (°)	116.320 (5), 98.878 (5), 98.390 (5)
*V* (Å^3^)	838.1 (3)
*Z*	2
Radiation type	Mo *K*α
μ (mm^−1^)	0.12
Crystal size (mm)	0.20 × 0.18 × 0.15

Data collection	
Diffractometer	Bruker Kappa APEXII
Absorption correction	Multi-scan (*SADABS*; Bruker, 2012 ▸)
*T*_min_, *T*_max_	0.834, 0.942
No. of measured, independent and observed [*I* > 2σ(*I*)] reflections	15411, 3265, 1835
*R*_int_	0.067
(sin θ/λ)_max_ (Å^−1^)	0.615

Refinement	
*R*[*F* ^2^ > 2σ(*F* ^2^)], *wR*(*F* ^2^), *S*	0.049, 0.133, 1.02
No. of reflections	3265
No. of parameters	264
No. of restraints	7
H-atom treatment	H atoms treated by a mixture of independent and constrained refinement
Δρ_max_, Δρ_min_ (e Å^−3^)	0.24, −0.21

Computer programs: *APEX2*, *SAINT* and *XPREP* (Bruker, 2012 ▸), *SHELXT2014/5* (Sheldrick, 2015*a* ▸), *SHELXL2018/3* (Sheldrick, 2015*b* ▸), *Mercury* (Macrae *et al.*, 2020 ▸) and *PLATON* (Spek, 2020 ▸).

## supplementary crystallographic information

### Crystal data

**Table:**

C_4_H_12_N_2_^2+^·C_7_H_2_N_2_O_7_^2^^−^·0.5C_4_H_10_N_2_·H_2_O	*Z* = 2
*M**_r_* = 375.35	*F*(000) = 396
Triclinic, *P*1	*D*_x_ = 1.487 Mg m^−^^3^
*a* = 6.6211 (16) Å	Mo *K*α radiation, λ = 0.71073 Å
*b* = 11.891 (3) Å	Cell parameters from 2314 reflections
*c* = 12.389 (3) Å	θ = 3.2–20.9°
α = 116.320 (5)°	µ = 0.12 mm^−^^1^
β = 98.878 (5)°	*T* = 296 K
γ = 98.390 (5)°	BLOCK, orange
*V* = 838.1 (3) Å^3^	0.20 × 0.18 × 0.15 mm

### Data collection

**Table:**

Bruker Kappa APEXII diffractometer	1835 reflections with *I* > 2σ(*I*)
Radiation source: fine-focus sealed tube	*R*_int_ = 0.067
ω and φ scan	θ_max_ = 25.9°, θ_min_ = 1.9°
Absorption correction: multi-scan (SADABS; Bruker, 2012)	*h* = −8→8
*T*_min_ = 0.834, *T*_max_ = 0.942	*k* = −14→14
15411 measured reflections	*l* = −15→15
3265 independent reflections	

### Refinement

**Table:**

Refinement on *F*^2^	Secondary atom site location: difference Fourier map
Least-squares matrix: full	Hydrogen site location: mixed
*R*[*F*^2^ > 2σ(*F*^2^)] = 0.049	H atoms treated by a mixture of independent and constrained refinement
*wR*(*F*^2^) = 0.133	*w* = 1/[σ^2^(*F*_o_^2^) + (0.0514*P*)^2^ + 0.1429*P*] where *P* = (*F*_o_^2^ + 2*F*_c_^2^)/3
*S* = 1.02	(Δ/σ)_max_ < 0.001
3265 reflections	Δρ_max_ = 0.24 e Å^−^^3^
264 parameters	Δρ_min_ = −0.21 e Å^−^^3^
7 restraints	Extinction correction: *SHELXL2018/3* (Sheldrick 2015b), Fc^*^=kFc[1+0.001xFc^2^λ^3^/sin(2θ)]^-1/4^
Primary atom site location: dual	Extinction coefficient: 0.022 (3)

### Special details

**Table:**

Geometry. All esds (except the esd in the dihedral angle between two l.s. planes) are estimated using the full covariance matrix. The cell esds are taken into account individually in the estimation of esds in distances, angles and torsion angles; correlations between esds in cell parameters are only used when they are defined by crystal symmetry. An approximate (isotropic) treatment of cell esds is used for estimating esds involving l.s. planes.

### Fractional atomic coordinates and isotropic or equivalent isotropic displacement parameters (Å^2^)

**Table:**

	*x*	*y*	*z*	*U*_iso_*/*U*_eq_	
C1	0.2400 (4)	0.4979 (2)	0.4846 (2)	0.0331 (6)	
C2	0.1716 (4)	0.5004 (2)	0.3755 (2)	0.0376 (6)	
H2	0.097021	0.424987	0.303896	0.045*	
C3	0.2150 (4)	0.6162 (3)	0.3737 (2)	0.0353 (6)	
C4	0.3382 (4)	0.7355 (2)	0.4796 (2)	0.0335 (6)	
C5	0.4064 (4)	0.7254 (2)	0.5918 (2)	0.0303 (6)	
C6	0.3534 (4)	0.6100 (2)	0.5917 (2)	0.0325 (6)	
H6	0.394113	0.606279	0.665144	0.039*	
C7	0.5309 (4)	0.8402 (2)	0.7123 (2)	0.0362 (6)	
C8	0.1947 (4)	0.0896 (2)	0.5817 (2)	0.0388 (7)	
H8A	0.166572	0.172139	0.595530	0.047*	
H8B	0.336846	0.105846	0.628741	0.047*	
C9	−0.0406 (4)	−0.0246 (3)	0.3735 (2)	0.0379 (7)	
H9A	−0.048896	−0.081723	0.286864	0.046*	
H9B	−0.077271	0.053723	0.379768	0.046*	
C10	0.4553 (5)	0.5527 (3)	−0.0810 (3)	0.0530 (8)	
H10A	0.593339	0.595334	−0.079123	0.064*	
H10B	0.351574	0.562706	−0.138468	0.064*	
C11	0.5558 (5)	0.5896 (3)	0.1283 (3)	0.0519 (8)	
H11A	0.517856	0.623969	0.207334	0.062*	
H11B	0.699858	0.634402	0.142542	0.062*	
C12	0.9027 (5)	1.0964 (3)	0.0763 (3)	0.0663 (9)	
H12A	0.991252	1.127416	0.158769	0.080*	
H12B	0.793302	1.143558	0.084060	0.080*	
C13	0.9676 (5)	0.8797 (3)	0.0037 (3)	0.0660 (9)	
H13A	0.899018	0.788492	−0.035061	0.079*	
H13B	1.058073	0.903815	0.083872	0.079*	
N1	0.1911 (3)	0.3784 (2)	0.4897 (3)	0.0448 (6)	
N2	0.1279 (4)	0.6133 (3)	0.2575 (2)	0.0498 (6)	
N3	0.1777 (3)	0.0079 (2)	0.4483 (2)	0.0366 (6)	
N4	0.4177 (4)	0.6159 (2)	0.0429 (2)	0.0492 (6)	
N5	0.8059 (4)	0.9568 (3)	0.0213 (2)	0.0594 (8)	
O1	0.2522 (3)	0.37951 (19)	0.5894 (2)	0.0588 (6)	
O2	0.0900 (3)	0.28024 (19)	0.3949 (2)	0.0618 (6)	
O3	0.0716 (3)	0.5083 (2)	0.16184 (19)	0.0659 (6)	
O4	0.1115 (4)	0.7148 (2)	0.25659 (19)	0.0693 (7)	
O5	0.5161 (3)	0.83365 (19)	0.80899 (17)	0.0578 (6)	
O6	0.6420 (3)	0.93523 (17)	0.71300 (16)	0.0481 (5)	
O7	0.3856 (3)	0.84047 (17)	0.47722 (17)	0.0499 (5)	
O8	0.5428 (4)	0.8759 (3)	0.1338 (2)	0.0634 (7)	
H8C	0.489 (6)	0.796 (4)	0.102 (3)	0.089 (13)*	
H8D	0.486 (6)	0.928 (4)	0.182 (4)	0.102 (16)*	
H3A	0.267 (4)	0.045 (2)	0.418 (3)	0.069 (10)*	
H3B	0.224 (4)	−0.0629 (19)	0.438 (2)	0.058 (9)*	
H4	0.291 (3)	0.582 (3)	0.039 (3)	0.061 (10)*	
H5A	0.726 (4)	0.943 (3)	0.069 (2)	0.086 (12)*	
H5B	0.712 (4)	0.927 (3)	−0.0537 (18)	0.083 (12)*	

### Atomic displacement parameters (Å^2^)

**Table:**

	*U*^11^	*U*^22^	*U*^33^	*U*^12^	*U*^13^	*U*^23^
C1	0.0281 (14)	0.0283 (14)	0.0472 (17)	0.0077 (11)	0.0161 (12)	0.0193 (13)
C2	0.0297 (14)	0.0347 (15)	0.0386 (16)	0.0071 (12)	0.0120 (12)	0.0082 (13)
C3	0.0316 (14)	0.0434 (17)	0.0343 (15)	0.0110 (12)	0.0105 (12)	0.0202 (14)
C4	0.0272 (14)	0.0344 (15)	0.0453 (16)	0.0096 (12)	0.0129 (12)	0.0227 (14)
C5	0.0288 (14)	0.0316 (14)	0.0349 (15)	0.0091 (11)	0.0129 (11)	0.0174 (13)
C6	0.0284 (13)	0.0352 (15)	0.0404 (16)	0.0104 (12)	0.0143 (12)	0.0209 (14)
C7	0.0346 (15)	0.0327 (15)	0.0416 (17)	0.0087 (12)	0.0139 (13)	0.0164 (14)
C8	0.0344 (15)	0.0385 (16)	0.0378 (17)	0.0012 (12)	0.0044 (12)	0.0170 (14)
C9	0.0399 (16)	0.0409 (16)	0.0346 (15)	0.0057 (12)	0.0073 (12)	0.0212 (14)
C10	0.057 (2)	0.0492 (19)	0.057 (2)	0.0128 (15)	0.0165 (16)	0.0281 (17)
C11	0.0501 (18)	0.0477 (19)	0.0454 (18)	0.0058 (15)	0.0065 (15)	0.0150 (16)
C12	0.054 (2)	0.068 (2)	0.052 (2)	0.0006 (17)	0.0126 (17)	0.0117 (18)
C13	0.053 (2)	0.071 (2)	0.067 (2)	−0.0030 (18)	0.0156 (18)	0.032 (2)
N1	0.0357 (14)	0.0330 (15)	0.0707 (19)	0.0119 (11)	0.0243 (13)	0.0244 (15)
N2	0.0439 (15)	0.0615 (18)	0.0459 (17)	0.0146 (13)	0.0126 (12)	0.0261 (16)
N3	0.0358 (13)	0.0362 (14)	0.0446 (15)	0.0078 (11)	0.0133 (11)	0.0241 (12)
N4	0.0466 (16)	0.0431 (15)	0.0527 (16)	0.0098 (13)	0.0149 (13)	0.0180 (13)
N5	0.0428 (16)	0.081 (2)	0.0382 (16)	−0.0074 (15)	0.0115 (14)	0.0208 (16)
O1	0.0646 (14)	0.0533 (14)	0.0814 (16)	0.0181 (11)	0.0241 (12)	0.0485 (13)
O2	0.0579 (14)	0.0295 (12)	0.0816 (16)	0.0017 (10)	0.0193 (12)	0.0146 (12)
O3	0.0694 (15)	0.0694 (16)	0.0389 (13)	0.0079 (12)	0.0052 (11)	0.0141 (12)
O4	0.0837 (17)	0.0753 (17)	0.0616 (15)	0.0283 (13)	0.0092 (12)	0.0435 (14)
O5	0.0679 (14)	0.0570 (13)	0.0370 (12)	−0.0114 (10)	0.0063 (10)	0.0226 (11)
O6	0.0558 (12)	0.0350 (11)	0.0451 (12)	−0.0015 (9)	0.0207 (9)	0.0130 (9)
O7	0.0409 (11)	0.0458 (12)	0.0705 (14)	0.0011 (9)	0.0020 (10)	0.0408 (11)
O8	0.0809 (18)	0.0400 (15)	0.0751 (18)	0.0151 (14)	0.0457 (14)	0.0240 (14)

### Geometric parameters (Å, º)

**Table:**

C1—C2	1.373 (3)	C10—H10A	0.9700
C1—C6	1.390 (3)	C10—H10B	0.9700
C1—N1	1.442 (3)	C11—N4	1.460 (3)
C2—C3	1.376 (3)	C11—H11A	0.9700
C2—H2	0.9300	C11—H11B	0.9700
C3—C4	1.443 (3)	C12—N5	1.478 (4)
C3—N2	1.448 (3)	C12—C13^iii^	1.497 (4)
C4—O7	1.257 (3)	C12—H12A	0.9700
C4—C5	1.455 (3)	C12—H12B	0.9700
C5—C6	1.366 (3)	C13—N5	1.488 (4)
C5—C7	1.503 (3)	C13—H13A	0.9700
C6—H6	0.9300	C13—H13B	0.9700
C7—O6	1.251 (3)	N1—O2	1.227 (3)
C7—O5	1.251 (3)	N1—O1	1.232 (3)
C8—N3	1.475 (3)	N2—O3	1.232 (3)
C8—C9^i^	1.507 (3)	N2—O4	1.233 (3)
C8—H8A	0.9700	N3—H3A	0.903 (16)
C8—H8B	0.9700	N3—H3B	0.900 (16)
C9—N3	1.484 (3)	N4—H4	0.860 (17)
C9—H9A	0.9700	N5—H5A	0.900 (17)
C9—H9B	0.9700	N5—H5B	0.918 (17)
C10—N4	1.461 (3)	O8—H8C	0.84 (4)
C10—C11^ii^	1.510 (4)	O8—H8D	0.84 (4)
			
C2—C1—C6	120.9 (2)	N4—C11—H11A	108.9
C2—C1—N1	120.0 (2)	C10^ii^—C11—H11A	108.9
C6—C1—N1	119.1 (2)	N4—C11—H11B	108.9
C1—C2—C3	119.0 (2)	C10^ii^—C11—H11B	108.9
C1—C2—H2	120.5	H11A—C11—H11B	107.7
C3—C2—H2	120.5	N5—C12—C13^iii^	110.2 (3)
C2—C3—C4	123.4 (2)	N5—C12—H12A	109.6
C2—C3—N2	116.1 (2)	C13^iii^—C12—H12A	109.6
C4—C3—N2	120.5 (2)	N5—C12—H12B	109.6
O7—C4—C3	123.7 (2)	C13^iii^—C12—H12B	109.6
O7—C4—C5	121.7 (2)	H12A—C12—H12B	108.1
C3—C4—C5	114.5 (2)	N5—C13—C12^iii^	110.0 (3)
C6—C5—C4	120.6 (2)	N5—C13—H13A	109.7
C6—C5—C7	117.2 (2)	C12^iii^—C13—H13A	109.7
C4—C5—C7	122.2 (2)	N5—C13—H13B	109.7
C5—C6—C1	121.6 (2)	C12^iii^—C13—H13B	109.7
C5—C6—H6	119.2	H13A—C13—H13B	108.2
C1—C6—H6	119.2	O2—N1—O1	122.8 (2)
O6—C7—O5	123.4 (2)	O2—N1—C1	118.6 (3)
O6—C7—C5	120.5 (2)	O1—N1—C1	118.5 (2)
O5—C7—C5	116.1 (2)	O3—N2—O4	121.8 (2)
N3—C8—C9^i^	110.7 (2)	O3—N2—C3	118.6 (2)
N3—C8—H8A	109.5	O4—N2—C3	119.7 (3)
C9^i^—C8—H8A	109.5	C8—N3—C9	111.2 (2)
N3—C8—H8B	109.5	C8—N3—H3A	113.0 (19)
C9^i^—C8—H8B	109.5	C9—N3—H3A	109.0 (18)
H8A—C8—H8B	108.1	C8—N3—H3B	108.8 (17)
N3—C9—C8^i^	110.8 (2)	C9—N3—H3B	112.4 (18)
N3—C9—H9A	109.5	H3A—N3—H3B	102.2 (19)
C8^i^—C9—H9A	109.5	C11—N4—C10	110.1 (2)
N3—C9—H9B	109.5	C11—N4—H4	106.5 (19)
C8^i^—C9—H9B	109.5	C10—N4—H4	108 (2)
H9A—C9—H9B	108.1	C12—N5—C13	111.6 (2)
N4—C10—C11^ii^	113.1 (2)	C12—N5—H5A	110 (2)
N4—C10—H10A	109.0	C13—N5—H5A	111 (2)
C11^ii^—C10—H10A	109.0	C12—N5—H5B	111 (2)
N4—C10—H10B	109.0	C13—N5—H5B	109 (2)
C11^ii^—C10—H10B	109.0	H5A—N5—H5B	104 (2)
H10A—C10—H10B	107.8	H8C—O8—H8D	119 (4)
N4—C11—C10^ii^	113.3 (2)		
			
C6—C1—C2—C3	−0.7 (4)	C4—C5—C7—O6	−25.2 (4)
N1—C1—C2—C3	178.2 (2)	C6—C5—C7—O5	−23.2 (3)
C1—C2—C3—C4	3.0 (4)	C4—C5—C7—O5	155.0 (2)
C1—C2—C3—N2	−176.6 (2)	C2—C1—N1—O2	0.7 (3)
C2—C3—C4—O7	177.0 (2)	C6—C1—N1—O2	179.6 (2)
N2—C3—C4—O7	−3.4 (4)	C2—C1—N1—O1	−179.0 (2)
C2—C3—C4—C5	−2.6 (3)	C6—C1—N1—O1	−0.1 (3)
N2—C3—C4—C5	177.0 (2)	C2—C3—N2—O3	−18.7 (3)
O7—C4—C5—C6	−179.7 (2)	C4—C3—N2—O3	161.7 (2)
C3—C4—C5—C6	−0.1 (3)	C2—C3—N2—O4	161.1 (2)
O7—C4—C5—C7	2.1 (4)	C4—C3—N2—O4	−18.5 (4)
C3—C4—C5—C7	−178.2 (2)	C9^i^—C8—N3—C9	56.3 (3)
C4—C5—C6—C1	2.3 (3)	C8^i^—C9—N3—C8	−56.4 (3)
C7—C5—C6—C1	−179.4 (2)	C10^ii^—C11—N4—C10	−52.3 (3)
C2—C1—C6—C5	−2.0 (4)	C11^ii^—C10—N4—C11	52.1 (3)
N1—C1—C6—C5	179.1 (2)	C13^iii^—C12—N5—C13	57.4 (4)
C6—C5—C7—O6	156.6 (2)	C12^iii^—C13—N5—C12	−57.4 (4)

Symmetry codes: (i) −*x*, −*y*, −*z*+1; (ii) −*x*+1, −*y*+1, −*z*; (iii) −*x*+2, −*y*+2, −*z*.

### Hydrogen-bond geometry (Å, º)

**Table:**

*D*—H···*A*	*D*—H	H···*A*	*D*···*A*	*D*—H···*A*
O8—H8*D*···O6^iv^	0.84 (4)	1.98 (4)	2.807 (3)	173 (4)
N3—H3*A*···O6^v^	0.90 (2)	1.92 (2)	2.752 (3)	153 (3)
N3—H3*A*···O7^v^	0.90 (2)	2.33 (3)	2.940 (3)	125 (2)
N3—H3*B*···O4^vi^	0.90 (2)	2.50 (2)	3.142 (3)	129 (2)
N3—H3*B*···O7^vi^	0.90 (2)	1.84 (2)	2.696 (3)	157 (3)
N4—H4···O3	0.860 (17)	2.59 (2)	3.304 (3)	141 (2)
N5—H5*A*···O8	0.900 (17)	1.83 (2)	2.712 (3)	166 (3)
N5—H5*B*···O5^vii^	0.918 (17)	1.74 (2)	2.637 (3)	165 (3)
O8—H8*C*···N4	0.84 (4)	1.88 (4)	2.717 (3)	169 (3)
C8—H8*A*···O1	0.97	2.49	3.369 (3)	151
C9—H9*B*···O2	0.97	2.67	3.482 (3)	141
C12—H12*A*···O2^viii^	0.97	2.58	3.455 (4)	151
C12—H12*B*···O5^iv^	0.97	2.59	3.353 (4)	136

Symmetry codes: (iv) −*x*+1, −*y*+2, −*z*+1; (v) −*x*+1, −*y*+1, −*z*+1; (vi) *x*, *y*−1, *z*; (vii) *x*, *y*, *z*−1; (viii) *x*+1, *y*+1, *z*.
